# Potential Antimigraine Effects of Warfarin: An Exploration of Biological Mechanism with Survey of Patients

**DOI:** 10.1055/s-0039-1692989

**Published:** 2019-06-21

**Authors:** Benjamin Nilsson, Valentina Back, Ran Wei, Frances Plane, Paul Jurasz, Tammy J. Bungard

**Affiliations:** 1 Alberta Health Services, Edmonton, Alberta, Canada; 2 Faculty of Pharmacy and Pharmaceutical Sciences, University of Alberta, Edmonton, Alberta, Canada; 3 Cardiovascular Research Centre, University of Alberta, Edmonton, Alberta, Canada; 4 Department of Pharmacology, Faculty of Medicine and Dentistry, University of Alberta, Edmonton, Alberta, Canada; 5 Alberta Mazankowski Heart Institute, University of Alberta, Edmonton, Alberta, Canada; 6 Department of Cardiology, Faculty of Medicine and Dentistry, University of Alberta, Edmonton, Alberta, Canada

**Keywords:** warfarin, migraine, platelets, serotonin, blood vessels

## Abstract

Case reports suggest a link between anticoagulant use and improved migraine symptoms, and a role for platelet-induced cerebral vasoconstriction in migraine pathobiology. Hence, we investigated the mechanism by which warfarin may affect migraine symptoms and whether there is a change in migraine symptomology in patients initiating oral anticoagulants, most commonly warfarin. The effects of warfarin on human platelet aggregation and secretion as well as platelet-induced rat cerebral artery vasoconstriction were studied. A survey of migraine and symptom change after starting or stopping oral anticoagulants was also conducted. Warfarin inhibited platelet aggregation and 5-hydroxytryptamine (5-HT) secretion in a concentration-dependent manner. Warfarin-inhibited platelet secretion products constricted middle cerebral arteries from male but not from female rats. For the survey, patient demographic information, migraine and medical history, and Migraine Disability Assessment Score (MIDAS) changes were collected. Out of 175 consenting, 40 respondents met the criteria for migraine and completed the survey. A total of 11 patients reported migraine symptom change, all coinciding with starting warfarin. Of those having symptom and MIDAS improvement, most were female with migraines with aura, whereas those worsening were male with fewer having migraine with aura. Of those reporting migraine symptom change with warfarin, female sex may be associated with improved MIDAS, and those experiencing an aura component are more likely to report a symptom change. Warfarin-mediated symptom improvement in females may occur due to inhibition of platelet 5-HT secretion and a lower sensitivity of female cerebral blood vessels to platelet-derived 5-HT-induced vasoconstriction.

## Introduction

The pathogenesis of migraine is unclear. Based on early reports, it was proposed that the aura with migraines (classical migraine) was caused by cerebral vasoconstriction that then proceeded to a reactive intracranial and extracranial vasodilation with the associated headache. 1 Investigations of cerebral blood flow have not fully supported this early theory, as transition from vasoconstriction to vasodilatory hyperemia does not necessarily coincide with the onset of headache and as the hyperemia may persist following headache disappearance. 2 3 Subsequently, it has been hypothesized that migraines occur due to neurovascular mechanisms that lead to dysfunction in neuronal and broad sensory processing due to activation of the trigeminovascular system and neurogenic inflammation. 4 In addition to the reported cerebral hemodynamic changes associated with migraine, it has been suggested that platelets may also be involved in migraine pathogenesis. 5 Several studies have demonstrated enhanced platelet aggregation and 5-hydroxytryptamine (5-HT) secretion in migraineurs. 6 7 8 9 5-HT is thought to play an important role in migraine pathogenesis, as it is capable of modulating both pain transmission and vascular tone. 10

Previous studies have also shown that antiplatelet medications can have a demonstrable effect on migraine symptoms. One chart review of hospitalized patients found that acetylsalicylic acid (ASA) reduces the number of migraine with aura episodes by up to six-fold, 11 while another showed that clopidogrel reduces migraine with aura following closure of persistent foramen ovale and atrial septal defects. 12 Similarly, several case reports have also associated an improvement in migraine symptomatology with the use of vitamin K antagonists (VKAs). 13 14 15 16 17 We recently reported a case wherein remission of migraines was maintained throughout 12 years of warfarin therapy, recurred when the female patient was switched to apixaban, and then again resolved with the reintroduction of warfarin. 18 As some anticoagulants have been shown to also have antiplatelet effects, 19 20 21 platelets and their ability to secrete 5-HT are an attractive potential mechanism linking the activity of anticoagulant medications to potential changes in migraine symptoms. In support of such a mechanistic link, we sought to investigate the effect of warfarin on platelet function, 5-HT release, and vascular tone to determine whether there is a biologically plausible mechanism linking anticoagulant use to relief of migraine symptoms, as well as to survey patients to determine whether any patients noticed a change in migraine symptomatology with alteration in oral anticoagulant therapy within an anticoagulation clinic (AC). To our knowledge, the assessment of change in migraine symptoms has not been assessed in a larger population taking VKAs, even though an association between migraine with aura and an increased risk of ischemic stroke has been identified, particularly in females. 22 23

## Methods

### Platelet Isolation and Aggregation

Approval for the study was obtained from the University of Alberta Research Ethics Board. Following informed consent, blood was collected from healthy volunteers who had not taken any drugs affecting platelet function for 14 days prior to the study. Prostacyclin (prostaglandin I2 [PGI _2_ ])-washed platelets (2.5 × 10 ^8^ /mL) were prepared in Tyrode's buffer, and platelet aggregation in response to collagen (0.6 μg/mL) was measured as light transmittance percentage in a lumi-aggregometer (Chronolog, Havertown, Pennsylvania, United States) as described previously. 24 The inhibitory effects of warfarin (0–30 μg/mL) on platelet aggregation were normalized and extent of aggregation expressed as percent of vehicle (saline) control. After aggregation, platelet releasates were separated from pellets by adding 1 μg/mL PGI _2_ to aggregated samples, followed by centrifugation (10,000 *g* for 5 minutes). The releasates were then stored at −80°C prior to further analysis.

### Enzyme-Linked Immunosorbent Assay

As markers of platelet α- and δ-granule secretion, vascular endothelial cell growth factor (VEGF _165_ ) and serotonin (5-HT) were quantified in platelet releasates using the Quantikine ELISA Kit for human VEGF (R&D Systems, Minneapolis, Minnesota, United States) and a Serotonin enzyme-linked immunosorbent assay (ELISA) kit (Enzo Life Sciences, Brockville, Ontario, Canada), respectively. ELISAs were performed according to manufacturer's instructions and absorbance measured using an iMark 96-well plate reader (Bio-Rad, Mississauga, Ontario, Canada). The effects of warfarin on platelet secretion of VEGF and 5-HT were then expressed as percent of vehicle (saline) control.

### Animal Care and Use

All animal care and experimental procedures were approved by the Animal Care and Use Committee at the University of Alberta and performed in accordance with Canadian Council on Animal Care guidelines. Male Sprague-Dawley rats (250–300 g; Bioscience, University of Alberta) were housed in an enriched environment maintained on a 12:12-hour light–dark cycle at approximately 23°C with fresh tap water and standard chow available ad libitum. Rats were euthanized by inhalation of isoflurane followed by decapitation. The middle cerebral arteries were removed postmortem and placed in cold Kreb's buffer containing (mM) NaCl 119.0, NaHCO _3_ 25.0, KCl 4.7, MgSO _4_ 1.2, KH _2_ PO _4_ 1.18, glucose 11, and CaCl _2_ 2.5.

### Pressure Myography

Leak-free segments of middle cerebral artery (MCA; 2–3 mm in length) were mounted between two glass cannulae in an arteriograph chamber (Living Systems Instrumentation, Burlington, Vermont, United States) under conditions of no luminal flow. Vessels were bathed in Kreb's buffer at 37°C gassed with 4.92% CO _2_ , 20.92% O _2_ , and balance N _2_ (pH 7.4) and intravascular pressure was maintained via a pressure servo-control system (PS200, Living Systems Instrumentation). Arteries were viewed through a Nikon TMS inverted microscope, and measurements of the internal diameter were made via an automated video dimension analyzer (VDA10, Living Systems Instrumentation). The glass cannulae (borosilicate glass with outer diameter of 1.2 mm and inner diameter of 0.69 mm) were pulled using a Model P87 Flaming/Brown micropipette puller (Sutter Instruments, Novato, California, United States). Pressure and diameter measurements were recorded via Powerlab data acquisition system using the data acquisition software LabChart 5 (AD Instruments, Colorado, United States). MCAs which did not develop myogenic tone during an initial equilibration period of 45 minutes at 80 mm Hg and 37°C were discarded. Following the equilibration period, the intravascular pressure was set to 40 mm Hg and held at that pressure for 15 minutes before the addition of human platelet releasate into the bath. Platelet releasate was in the bath for a minimum of 15 minutes. In some experiments, ketanserin (1 μM) was added to the bath 15 minutes prior to the addition of the platelet releasate. In separate experiments, arterial responsiveness to 5-HT was assessed by cumulative additions of 5-HT (1 nM to 3 μM) into the bath. The effect of releasates and 5-HT on arterial diameter was measured as the change in baseline in microns (μm).

### Survey

#### Population and Survey Conduction

Survey ethics approval was received through the Research Ethics Board at the University of Alberta. Eligible patients had their warfarin managed by the AC at the University of Alberta Hospital, Edmonton, Alberta, Canada, that manages approximately 750 patients. Participants had to be older than 18 years, have initiated the anticoagulant within 10 years, have administered the anticoagulant for the past 3 months, and have access to the Internet and be able to speak/read English. Over the course of 4 weeks, the AC staff screened and approached all contacted patients using a standardized script to participate in the survey (regardless of an awareness of migraine history) and collected email addresses for those agreeing. Four weeks was deemed an appropriate time interval given the AC recalls patients (at the longest) on a monthly basis. The survey was constructed using the Qualtrics software platform. Links were sent out to agreeable patients with reminders occurring at 2 and 4 weeks and the survey remained open for a total of 5 weeks. Implied consent was obtained with completion of the survey.

#### The Survey

The survey was divided into four sections: demographics, migraine history, anticoagulant use, and changes in migraine symptomatology associated with anticoagulant use. To have a history of migraines (and proceed with the survey), participants had to either identify an established medical diagnosis or at least 1 symptom among a list of 19 symptoms included based on the International Headache Society (IHS) Classification ICHD-11 criteria for migraine headache. 25 Anticoagulant usage, including start and stop times, was identified. Finally, alteration in migraine symptomatology in relation to anticoagulant use was determined; if a change in symptoms was apparent, further information was collected using the Migraine Disability Assessment Score (MIDAS). 25 The MIDAS assesses the impact migraines have on the daily level of pain and disability and contains the following: Grade 1 (score 0–5), little or no disability; Grade 2 (score 6–10), mild disability; Grade 3 (score 11–20), moderate disability; and Grade 4 (score 21+), severe disability. A reduction in MIDAS over time demonstrates improvement, whereas an increase demonstrates worsening of migraine symptoms. In qualifying changes in migraine symptomatology via the MIDAS, participants were asked to focus on the 3 months prior and 3 months after the anticoagulant change. Both prophylactic and acute and migraine therapies were also collected.

#### Survey Data Analysis

Among those having migraines, data were separated based on whether symptom change did or did not occur with alteration in oral anticoagulant. For those reporting symptom change, further breakdown was performed based on the change to the MIDAS (improved, worsened, or not changed) and the data were described.

#### Statistics

Statistics were performed using GraphPad Prism 7.0 software. All means are reported with standard error of the mean. One-way analysis of variance (ANOVA) with Dunnett's post hoc test and two-way ANOVA were performed where appropriate. A *p* -value < 0.05 was considered significant.

## Results

**Fig. 1:**
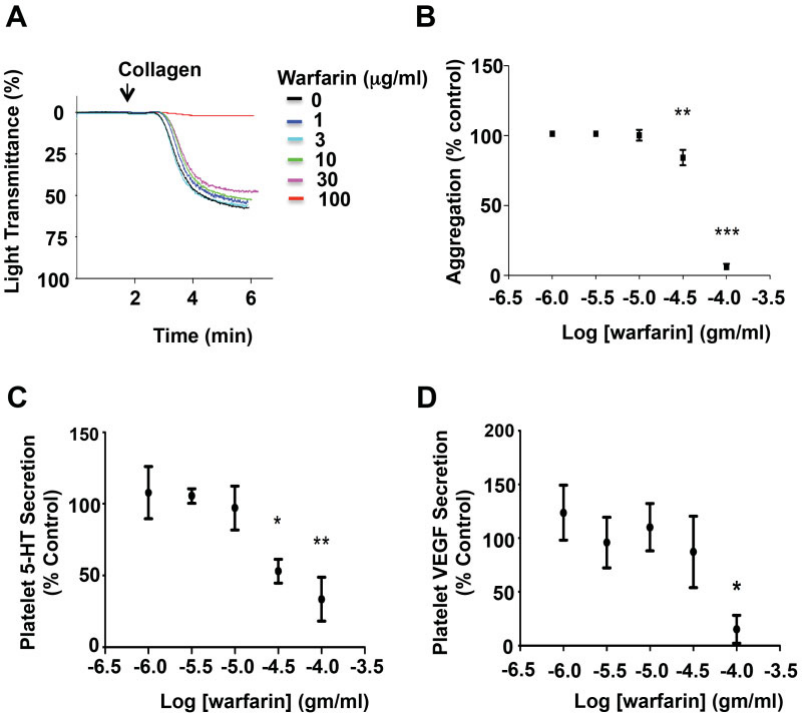
( **A** ) Representative platelet aggregometry traces and ( **B** ) summary data demonstrating the concentration-dependent inhibitory effects of warfarin on platelet aggregation. *N* = 5 (3 female donors and 2 male donors). ** *p* < 0.01; *** *p* < 0.001. ( **C, D** ) Summary data demonstrating the inhibitory effects of warfarin on platelet 5-HT and VEGF secretion, respectively. *N* = 5 (3 female donors and 2 male donors). * *p* < 0.05; ** *p* < 0.01. VEGF, vascular endothelial growth factor; 5-HT, 5-hydroxytryptamine.

We investigated the effects of warfarin on platelet aggregation and secretion as a possible mechanistic link between anticoagulant use and change in migraine symptoms. Warfarin inhibited platelet aggregation in response to collagen (0.6 μg/mL) in a concentration-dependent manner ( Fig. 1A ); and at 30 μg/mL it caused a small but significant inhibition in aggregation (84.3 ± 5.5% vs. 100% control aggregation; *p* < 0.05; Fig. 1B ). Interestingly, at 30 μg/mL warfarin had a large inhibitory effect on platelet 5-HT secretion in response to collagen (53.0 ± 8.4% vs. 100% control 5-HT secretion; *p* < 0.05). An uncoupling of 5-HT secretion inhibition from inhibition of platelet aggregation by warfarin was evident from the IC _50_ of the two events (32.9 vs. 69.0 μg/mL; Fig. 1C ). This type of uncoupling was not evident between inhibition of platelet aggregation and platelet VEGF secretion, as only 100 μg/mL warfarin significantly inhibited VEGF secretion ( Fig. 1D ).

**Fig. 2:**
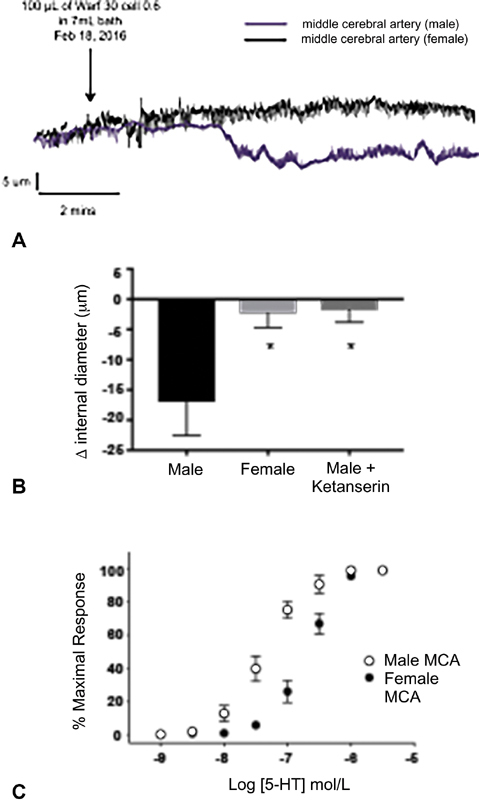
( **A** ) Representative pressure myography traces and ( **B** ) summary data demonstrating the 5-HT-dependent constrictive effects of warfarin (30 μg/mL) inhibited platelet releasates on male, but not on female, rat middle cerebral arteries. *N* = 4 for each sex. * *p* < 0.05. ( **C** ) Concentration responses experiments demonstrating vasoconstriction to 5-HT of male versus female rat MCAs. *N* = 5 for each sex. 5-HT, 5-hydroxytryptamine.

Previous case reports suggest that a sex difference may exist in migraine symptomology change with VKA therapy 13 14 15 16 17 18 ; however, no significant difference was observed in the effects of warfarin on 5-HT secretion from female versus male platelets. Therefore, to investigate potential differential effects of warfarin-inhibited platelet secretion on cerebral vascular tone in males versus females, pressure myography was performed on MCAs excised from male and female rats and treated with warfarin (30 μg/mL)-inhibited platelet releasates. Warfarin-inhibited platelet releasates caused a significantly greater constriction of male versus female MCAs (−16.99 ± 5.57 μm vs. −2.30 ± 2.41 μm change in internal diameter; *p* < 0.05; Fig. 2A ). This constriction of male MCAs by warfarin-inhibited platelet releasates occurred in response to platelet-secreted 5-HT as the 5-HT _2/1_ receptor antagonist Ketanserin inhibited the constriction of male MCAs in response to platelet releasates (−16.99 ± 5.57 μm vs. −1.82 ± 1.96 μm change in internal diameter; *p* < 0.05; Fig. 2B ). Consistent with this finding, concentration response experiments demonstrated that MCAs from male rats are more sensitive to the vasoconstrictive effects of 5-HT than those from females (EC _50_ of 42.1 vs. 202.7 nM; Fig. 2C ).

**Fig. 3:**
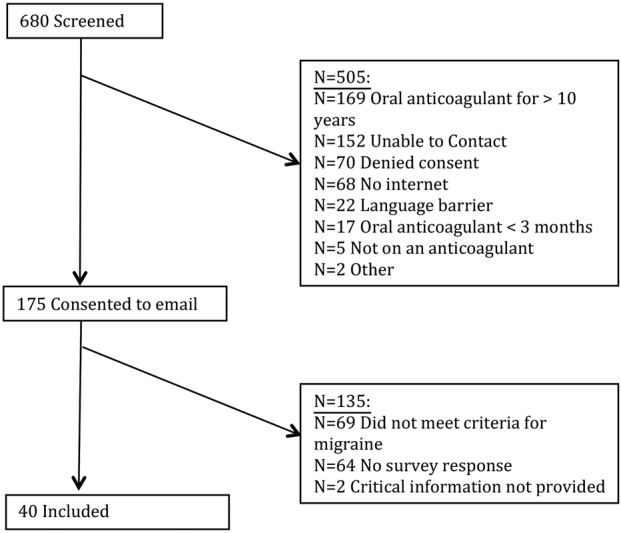
Patient flow of survey.

To determine whether our experimental findings could potentially explain changes in migraine symptomology with VKA therapy in the clinic, we screened 680 patients for inclusion into our survey of migraine and symptom change after starting or stopping oral anticoagulants. A total of 175 consented to receive the survey, with 40 patients included ( Fig. 3 ). Of the 40 with migraines, 11 (27.5%) reported a change in migraine severity, frequency or duration, and all reported this change upon starting warfarin therapy. Compared with those not reporting a change in migraine status, those with a change tended to be younger (with 41.3 and 72.7% being ≤ 55 years, respectively) and have migraine with aura (20.7 vs. 72.7%; Table 1 ). Both those that did not and did report a change in migraine status had several years since the onset of migraines (32 and 21 years), most commonly had warfarin indicated due to a mechanical valve (72.4 and 90.9%), and were taking warfarin for a median of 5 and 3 years, respectively.

**Table 1:** Demographics

Factor	Change in migraine symptoms, *N* = 11	No change in migraine symptoms, *N* = 29
Age		
< 36 y	2	2
36–45 y	1	4
46–55 y	5	6
56–65 y	1	11
> 65 y	2	6
Gender		
Female	4	15
Male	7	14
Type of migraine		
With aura	8	6
Without aura	1	9
Diagnosis only	2	14
Years since onset of migraine (median, IQR)	21 (10–29)	32 (21–41)
Duration of anticoagulant (median, IQR)	3 (1–6)	5 (1–8)
Anticoagulant		
Warfarin	11	27
Dabigatran	0	1
Rivaroxaban	0	1
Indication for anticoagulant a		
Mechanical valve	10	21
Atrial fibrillation	1	7
Venous thromboembolism	0	1
Stroke	0	2
Other	1 b	2 c
Medical history		
Congenital heart disease	7	16
Hypertension	4	14
Hyperlipidemia	1	8
Diabetes	1	7
Heart failure	2	7
Stroke/TIA	1	5
Myocardial infarction	1	1
Current smoker	0	2
Past smoker	4	15
Chronic daily ASA use with anticoagulant	4	11

Abbreviations: ASA, acetyl salicylic acid; IQR, interquartile range; TIA, transient ischemic attack.

a Not mutually exclusive.

b One portal vein thrombosis.

c One cerebral venous sinus thrombosis and one portal vein thrombosis.

Among the 11 reporting a change in their migraines, assessment of this change with the MIDAS identified an improvement for 5, worsening for 4, and no change for the remaining 2 ( Table 2 ). The five with both symptom report and MIDAS change reflecting improvement had substantial changes in the MIDAS (−19 [−24, −8]), with four reporting stopping acute migraine treatments. Notably, the majority with improvement were female (4/5), had migraine with aura (4/5), and had a history of smoking (3/5). Those having worsening of symptoms and MIDAS change reflecting this ( *N* = 4) also had substantial changes in the MIDAS (24 (14, 40)), with one patient reporting initiation of an acute migraine treatment. In contrast to those with improvements, those with symptom worsening were all male (4/4), with 50% having migraine with aura.

**Table 2:** Characterization based on change in MIDAS

Factor	Δ− MIDAS *N* = 5	Δ+ MIDAS *N* = 4	No Δ MIDAS *N* = 2
Median change in MIDAS (range)	−19 (−24 to −8)	24 (14–40)	–
Gender			
Female	4	0	0
Male	1	4	2
Type of migraine			
With aura	4	2	2
Without aura	0	1	0
Diagnosis only	1	1	0
Smoking status—past	3	1	0
Indication for anticoagulant			
Mechanical valve	4	4	2
Atrial fibrillation	1	0	0
Venous thromboembolism	0	0	0
Stroke	0	0	0
Medical history			
Congenital heart disease	3	3	1
Hypertension	3	0	1
Hyperlipidemia	1	0	0
Diabetes	0	0	1
Heart failure	0	2	0
Stroke	0	1	0
Myocardial infarction	0	0	1

Abbreviation: MIDAS, Migraine Disability Assessment Score.

Note: A reduction (Δ−) in MIDAS demonstrates an improvement, whereas an increase (Δ+) demonstrates worsening of migraine symptoms.

## Discussion

Whether a potential link exists between the anticoagulant effect of warfarin and resolution of migraines is unknown. While there are two cases reporting improvement in symptoms without therapeutic international normalized ratios (INRs; one within 2–3 days of warfarin initiation 18 and one while on a later warfarin dose reduced by 50% with INRs of 1.0–1.2), 13 a randomized open crossover study of acenocoumarol or propranolol identified only 1/12 to respond to low-intensity VKA (INR: 1.5–2.0). 26 Moreover, a patient changing from warfarin to a direct factor X inhibitor (apixaban) noted recurrence of migraine symptomatology with the therapy switch, implying the mechanism of improvement in migraine symptomatology is beyond a direct anticoagulant effect. 18 In search of a potential non-anticoagulant mechanistic link between warfarin and an apparent improvement in migraine symptomology, we focused on platelets and their ability to secrete 5-HT, as evidence demonstrates that platelet-derived 5-HT may also be involved in migraine pathogenesis. 5

The precise antiplatelet mechanism of action of warfarin is unclear. However, our data suggest that warfarin may preferentially inhibit platelet 5-HT release over that of aggregation, and that reduced platelet 5-HT secretion falls below the concentration threshold necessary for triggering vasoconstriction of female cerebral vessels, but not of males. Several previous studies have shown that coumarins and other benzopyrones may inhibit aggregation via several mechanisms including inhibiting cyclooxygenase-1 (COX-1)/thromboxane A _2_ synthesis, thromboxane A _2_ receptors, and by increasing platelet cAMP levels and nitric oxide production. 19 20 21 27 Alternatively, warfarin may inhibit the γ-carboxylation of platelet-derived growth arrest-specific 6 (Gas6) protein. 28 Inhibition of this vitamin K–dependent protein is known to dampen aggregation and secretion responses to platelet agonists. 29 The concentration of warfarin required to inhibit platelet aggregation and 5-HT secretion in our experiments was approximately 10-fold higher than the low micromolar concentrations that are achieved in vivo. 30 31 However, warfarin is extensively metabolized by the liver via cytochrome P450 enzymes into hydroxylated metabolites, 32 and hydroxycoumarins have been demonstrated to be starting compounds for the synthesis of derivatives with platelet aggregation inhibitory activity in low micromolar ranges. 27 33 Hence, in vivo, warfarin may be efficiently metabolized by the liver into metabolites with platelet inhibitory properties. Such metabolites may potentially account for warfarin's occasionally reported, albeit weak, antiplatelet effect in vivo 34 35 and the need for high warfarin concentrations to produce an antiplatelet effect in vitro (due to less efficient warfarin metabolism by platelets). Interestingly, warfarin had a greater inhibitory effect on platelet 5-HT secretion than aggregation. Pharmacological uncoupling of secretion from platelet aggregation has been previously described for selective inhibitors of protein kinase Cα and inhibitors of actin polymerization, although inhibition of α-granule secretion was described. 24 36 The ability of warfarin or its metabolites to preferentially inhibit platelet secretion over aggregation suggests it may impact components of platelet exocytotic machinery, such as soluble NSF attachment protein receptor (SNARE) proteins or their regulators. Interestingly, warfarin appears to preferentially inhibit δ-granule content secretion (5-HT) over that of the α-granules as measured by VEGF. This aspect may contribute to its beneficial effect in improving migraine symptomology in some patients, as 5-HT is a potent vasoconstrictor of human cerebral arteries via the 5-HT _1B_ receptor, 37 while VEGF is known for its vasodilatory effects. 38 In addition to warfarin, antiplatelet drugs that also inhibit platelet 5-HT secretion would also be expected to have a similar beneficial antimigraine effect as has been shown in the context of arterial shunt closure with a combination of clopidogrel and ASA. 12

Several imaging studies have previously reported cerebral hypoperfusion to accompany aura symptoms suggesting involvement of cerebral vasoconstriction, 2 3 while in our survey those participants reporting an improvement in migraine symptoms were female patients having migraine with aura. Therefore, we investigated the effect of warfarin-inhibited platelet releasates on vasoconstriction of rat female versus male MCAs to help experimentally explain these apparent sex differences in migraine symptom changes. In response to warfarin-inhibited platelet releasates, which contained reduced 5-HT concentrations, MCAs from male rats demonstrated an eight-fold greater constrictive response than those from female rats. This effect was solely attributed to 5-HT in the platelet releasates as the selective 5-HT _2/1_ receptor antagonist ketanserin abolished constriction of male rat MCAs and concentration response experiments confirmed increased sensitivity of male versus female MCAs to the vasoconstrictive effects of 5-HT. This finding is consistent with a previous report demonstrating increased sensitivity of human male versus female cerebral arteries to vasoconstrictor agonists. 39 Moreover, it has been reported that during attacks of migraine with aura, the initial hypoperfusion accompanying aura is followed by cerebral hyperperfusion, indicative of reactive vasodilation, during the headache phase. 3 40 The lack of an initial vasoconstrictive response by female cerebral arteries to the reduced concentrations of 5-HT found in warfarin-inhibited platelet releasates may explain the reported improvement in migraine symptomology upon warfarin initiation by female participants with migraine with aura in our study. Among those surveyed, the majority reporting an improvement in migraine symptoms and MIDAS were female patients having migraine with aura and positive smoking history and tended to be of younger age. In contrast, those reporting a worsening in migraine symptomatology were male with half reporting migraines with aura. Notably, among general comments in our survey, three patients reported still having the aura, although had either resolution or an improvement of migraines. The improvement seen with warfarin among those with migraine with aura is interesting in that these patients are also at greater risk of stroke compared with those without aura. 17

While our survey sought to assess changes in migraine symptomatology that encompassed both an improvement and worsening, others have assessed improvement alone. A Spanish survey of patients assessing improvement in migraine symptomatology (defined as a reduction in frequency by at least 60%) with acenocoumarol reported this among 63% (42/66) of patients. 41 Those improving were significantly more likely to report vomiting and having severe migraines relative to those reporting no improvement in migraines. It is notable that these authors correlate the clinical improvement in migraines with the severity of attacks. In our study, the resultant change in the MIDAS was also clinically significant in that the change in scores would result in a change in the grade of disability. The magnitude of impact of clinical improvement with VKA administration is further substantiated by case reports of patients wanting to stay on warfarin, despite having no further indication. 17 42

The general patient characteristics (age, gender, and migraine with aura) among those showing improvement in migraine symptomatology in our study are consistent with other literature reports. Of the 16 case reports of an improvement in migraine symptomatology with VKA therapy, 13 were less than 50 years of age (range of 24–46 years) 15 17 26 42 43 with the remaining 3 being 55, 68, and 71 years of age. 13 14 18 Only one of these cases was male. 15 Among the eight reports that provide a description of the migraine, seven cases report improvement and were identified as having migraine with aura. 13 14 15 17 18 42 43 While not all cases describe specific symptomatology changes, a few cases describe the ongoing presence of an aura with resolution/improvement in migraine severity/frequency, 15 17 as reported in our survey.

As younger age was also associated with improvement in migraine symptomology among these participants, sex hormones may play a major role in this warfarin-mediated improvement. Estrogen, well known for its vasodilatory and vascular protective function, 44 45 may counteract the constrictive effects of 5-HT in younger female vessels. Hence, a warfarin-induced reduction in platelet 5-HT secretion coupled with a reduced response to low concentrations of 5-HT by estrogen-protected female vessels may be responsible for the improvement in symptomology. Reduced platelet 5-HT secretion by warfarin may also improve migraine symptomology beyond a vascular mechanism, as 5-HT also plays important roles in nociception and neurogenic/neurovascular inflammation. 46 47

Our study has a few limitations. Specific to the patient survey, we are subject to both selection bias in that patients having a change in migraine symptoms with warfarin initiation may have been more likely to respond to our survey. Recall bias may have also occurred, as we did recruit patients who had started an oral anticoagulant as long as 10 years ago. However, selection and recall bias would be expected to affect migraine patients with and without aura as well as females and males similarly. In our study, it is worth noting that patients reporting a change in migraine symptoms were predominantly those with an aura component, and generally female and male patients reported opposite responses (improvement in migraine symptoms vs. worsening), suggesting survey bias did not influence our results. Further support for a causal relationship between improvement in migraine symptomatology and VKA use are the general consistencies in the timing of symptom change as well as cases of re-challenge that support an effect. While our study had patients focus on changes within 3 months of anticoagulant change, others have reported more specific timing of symptom change. While the timing of change is dependent on an individual patient's baseline frequency of migraine occurrence, one case reported an improvement in 2 to 3 days, 18 with others reported changes within a month. 13 14 17 42 Similarly, for those discontinuing a VKA, symptom recurrence is reported within 10 days to a month. 13 15 18 42 In terms of re-challenge with the VKA to discern consistency of change, three case reports have demonstrated this 14 15 18 and one double-blind trial in a patient administering either placebo for 8 weeks (2–3 migraines per week) or warfarin targeted to an INR of 2.0 to 3.0 (migraine resolution). 13 Following the phase wherein the INR was targeted to 2.0 to 3.0 (administration of warfarin 6 mg daily), a warfarin dose reduction to 3 mg daily (yielding INRs between 1 and 1.2) maintained the resolution of migraines.

Another limitation of our study is that MIDAS is a clinical assessment tool to detect differences in level of disability and not necessarily the symptomatology of a migraine. Two patients reported a change in their migraine symptomatology without a resultant change in their MIDAS, and we are not able to discern (based on survey design) if the symptom change was an improvement or worsening. Literature indicates that clinical improvement in migraines with VKA use is correlated with the severity of the attacks (survey) 41 42 perhaps, suggesting the symptom change occurring in these patients may have been less severe migraine symptomatology. Similarly, there are several limitations to our mechanistic ex vivo experiments including utilizing healthy donor platelets and rat MCAs, while also strictly focusing on platelet and vascular aspects of migraine pathophysiology. Nonetheless, our mechanistic experiments may be a starting point for animal model studies that may lead to development of novel VKA-dependent therapies for migraine with aura.

Finally, as patients with migraine with aura are at greater risk of ischemic stroke and venous thromboembolism than those without aura, 17 48 49 warfarin may be the anticoagulant of choice in these female patients providing both migraine relief and thrombotic protection.
